# Issues and Controversies in the Evolution of Oral Rehydration Therapy (ORT)

**DOI:** 10.3390/tropicalmed6010034

**Published:** 2021-03-12

**Authors:** David Nalin

**Affiliations:** Albany Medical College, Albany, NY 12208-3478, USA; nalindavid@gmail.com

**Keywords:** cholera, non-cholera acute watery diarrheas (AWDs), oral rehydration solutions (ORS), ORS formulations, sodium balance, hyponatremia, hyponatremic seizures, hyponatremic sequelae

## Abstract

The original studies demonstrating the efficacy of oral glucose-electrolytes solutions in reducing or eliminating the need for intravenous therapy to correct dehydration caused by acute watery diarrheas (AWD) were focused chiefly on cholera patients. Later research adapted the oral therapy (ORT) methodology for treatment of non-cholera AWDs including for pediatric patients. These adaptations included the 2:1 regimen using 2 parts of the original WHO oral rehydration solution (ORS) formulation followed by 1 part additional plain water, and a “low sodium” packet formulation with similar average electrolyte and glucose concentrations when dissolved in the recommended volume of water. The programmatic desire for a single ORS packet formulation has led to controversy over use of the “low sodium” formulations to treat cholera patients. This is the subject of the current review, with the conclusion that use of the low-sodium ORS to treat cholera patients leads to negative sodium balance, leading to hyponatremia and, in severe cases, particularly in pediatric cholera, to seizures and other complications of sodium depletion. Therefore it is recommended that two separate ORS packet formulations be used, one for cholera therapy and the other for non-cholera pediatric AWD.

## 1. Introduction

Since 1990, controversies in the field of oral rehydration therapy (ORT) have arisen concerning efforts to preserve a single formulation for cholera and non-cholera acute watery diarrheas (AWD) in patients of all ages, while modifying the original WHO (Oralyte) oral rehydration solution (ORS) formulation containing 90 mEq/L Na^+^, to address three goals: (1) to safely provide effective rehydration and maintenance therapy for both cholera and non-cholera AWDs; (2) to reduce duration and volume of diarrhea and (3) to reduce the need for restarting intravenous fluids after completing rehydration, during the oral maintenance period [1,2]. The treatment of dysentery and inflammatory diarrheal diseases in which dehydration is not the main focus of therapy is beyond the scope of this report.

This paper will offer a critical review of the major studies done to support the above goals, focusing on those studies chosen for the 2011 Cochrane review [3,4,5,6,7,8,9,10]. The Cochrane-reviewed studies, and others referenced in this review, dealt mainly with patients seen in hospital settings in a research context who had severe dehydration due to profuse adult or pediatric cholera and/or non-cholera AWD. It is chiefly in such patients that otherwise academic discussions of different ORS formulations and ORT methods translate into significantly different clinical outcomes. Optimal treatment of hospitalized patients with severe dehydration due to diarrhea requires measurement of intake and output using essential equipment such as Watten cholera cots [11] and ample supplies of appropriate I.V. and oral replacement fluids. Home or outpatient therapy of less severely dehydrated patients requires use chiefly of clinical signs of hydration status to monitor therapeutic status, and is best addressed in detail in a separate review.

The Cochrane review covered structural aspects of the studies, but did not comment in detail on the quality of the design, clinical research methods or analytic approach to the data, aspects of which will be the subject of this report. This review will also provide context by considering the fundamentals of ORT and ORS composition and the history of ORS evolution, special characteristics of cholera pathophysiology, limitations of prior studies and implications for future safety studies. 

## 2. Basic Principles of Oral Therapy Methodology

Oral rehydration and maintenance therapy for significantly dehydrating AWD is not a magic bullet which works simply by giving patients ORS to drink; achieving optimal results requires administering the oral solutions according to the following well established ORT methodologic principles. Effective and safe ORT rests on the basic principle of AWD treatment: *timely replacement of the water and electrolyte losses of AWD with matching volumes of an absorbable ORS with electrolyte content sufficient to replace the electrolyte losses of AWD.* Deviation from this principle has resulted either in higher ORT failure rates with reversion to I.V. therapy and/or electrolyte abnormalities with potentially serious and avoidable sequelae of severe hyponatrema or hypernatremia.

Gross diarrhea rate (GDR), or the volume of diarrheal stool passed over a given unit of time, is far less important for successful ORT and optimal success rates than net gut balance, or the difference between diarrheal volume and volume of ORS intake in a given observation period. An excess of ORS intake volume over diarrheal volume during a given observation period, usually 4 or 6 h, is called *positive net gut balance (PNGB)*, and early achievement of PNGB is key to successful ORT [12]. Moderate increases in *GDR* are of negligible consequence if exceeded by ORS intake.

Initial diarrhea rates after hospital admission for AWD determine subsequent total stool volume [13]. Two methods of prestratification of patients entered in research studies ensure validity of comparison groups: entry only of patients in shock due to dehydration, or, more elegantly, allocating patients after confirming comparable GDR rates during the several hours required for initial intravenous rehydration. Allocation based on any other criteria often leads to an imbalance in disease severity between comparison groups which can bias the outcomes and accounts for a significant amount of variability seen between different results from the different centers conducting these studies.

When severely dehydrated patients arrive at treatment centers for rehydration, they are generally at or past their peak diarrhea rate. At this point vomiting, sometimes massive at disease onset (e.g., cholera [14]), is waning, and may wane more rapidly with correction of acidosis using ORS or, if patients are in shock due to dehydration, using I.V. fluids containing bicarbonate or a base precursor [15]. Vomiting quickly subsides soon after initiating treatment of most patients arriving at treatment centers, and measured vomitus volumes are generally small in relation to diarrhea volumes in most patients. Failure to correct acidosis in severely dehydrated patients leads to increased risk of pulmonary edema during I.V. rehydration [16].

Maintaining PNGB using proper ORT methodology will allow sufficient net fluid absorption to replace insensible losses and, in addition, promote sufficient net absorption to permit use of ORS with sodium content modestly lower than that of diarrhea fluid (e.g., 120 mEq/L. in cholera patients), while maintaining positive electrolyte balance [17].

If inadequate oral intake results in delayed or failed achievement of PNGB, additional I.V. fluid will be needed. This can arise rarely from excess vomiting or more commonly from inadequate monitoring or supervision, leading to failure of pediatric patients to be given or to drink sufficient ORS. Also, pediatric diarrhea patients in endemic areas not uncommonly have malabsorption of glucose and dietary sugars [18,19] and will be at higher risk of ORT failure and hypernatremia due to excess water loss in diarrheal stools. Caregivers should be alert to such patients, who may require I.V. therapy, though they can respond to ORS formulations with glycine replacing glucose. Researchers must be careful to avoid disproportionately allocating such patients among comparison groups, to avoid confounding interpretation of outcome results.

Claims have been made, without presentation of objective quantitative evidence, that mothers are aware of gross stool volume. In the writer’s opinion, based on extensive experience, mothers are aware of time since onset and of subsequent duration of diarrhea, and of course would prefer to see it stop if asked; but this is different from perception of the small difference in stool volume measured in most studies of modified ORS formulations [20].

Even trained clinicians are unable to accurately guess diarrheal volumes without stool volume measurements, as shown when they were asked to guess the volume of synthetic diarrhea fluid tossed onto a bedsheet. Estimates were wildly inaccurate (Dr. Norbert Hirschhorn, personal communication). Mothers (and clinicians) would doubtless like to have available a drug which could stop the diarrhea in minutes, but no currently available ORS formulation does that.

Experience from many studies have confirmed that the taste and appearance of plain ORS do not influence ORS acceptability among moderately and severely dehydrated patients, consistent with a recent report regarding milder cases [21]. Sweetening the ORS has had a negative effect [22]. The enormous benefits of ORS result from preventing and quickly correcting signs of dehydration, which parents fully appreciate.

## 3. Home Therapy with the WHO 90 ORS Using the 2:1 Regimen

**Figure 1:**
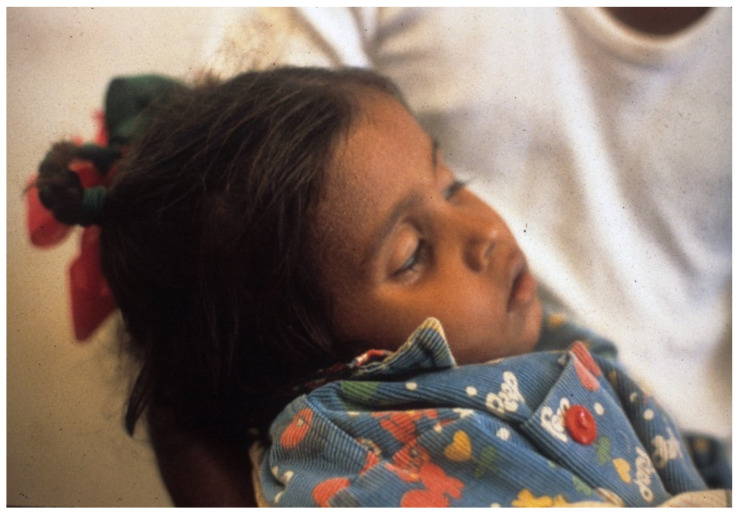
Severely dehydrated child in Greentown, Lahore, Pakistan. Note deeply sunken eyes and obtunded appearance. Etiology unknown.

**Figure 2:**
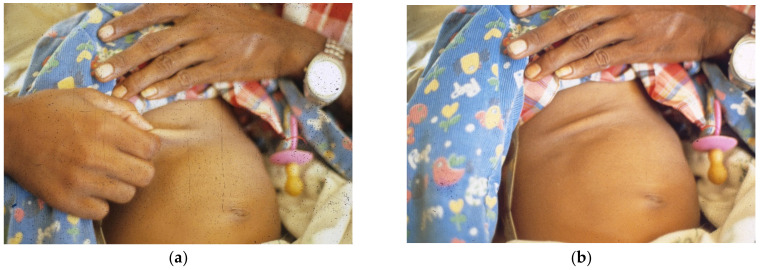
(**a**) Father pinches abdominal skin as instructed, (**b**) showing tenting indicating decreased elasticity after withdrawing hand.

**Figure 3:**
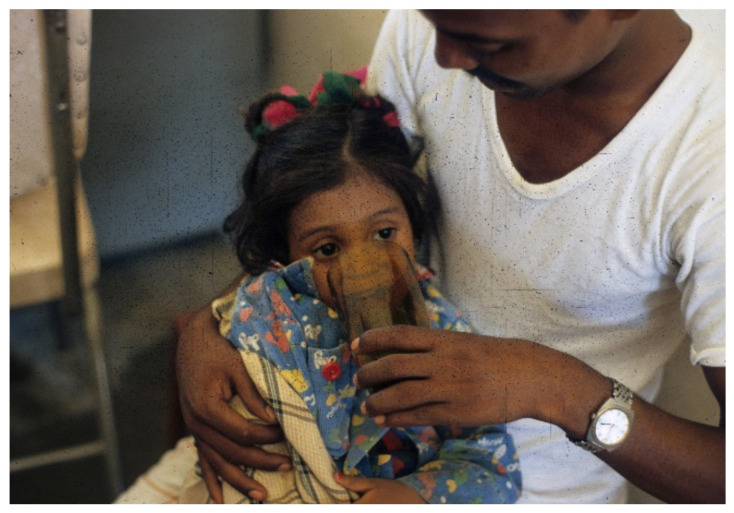
Father begins to offer patient oral rehydration solution (ORS) (WHO 90 formulation) to drink.

**Figure 4:**
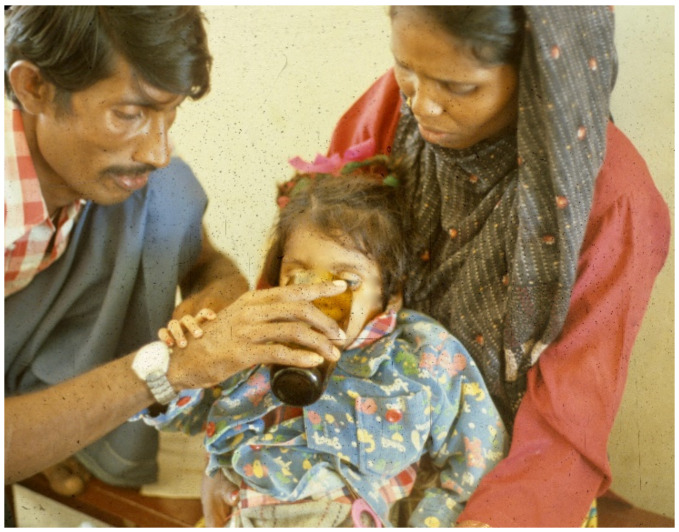
Patient continues to drink, using hand to keep ORS coming.

**Figure 5:**
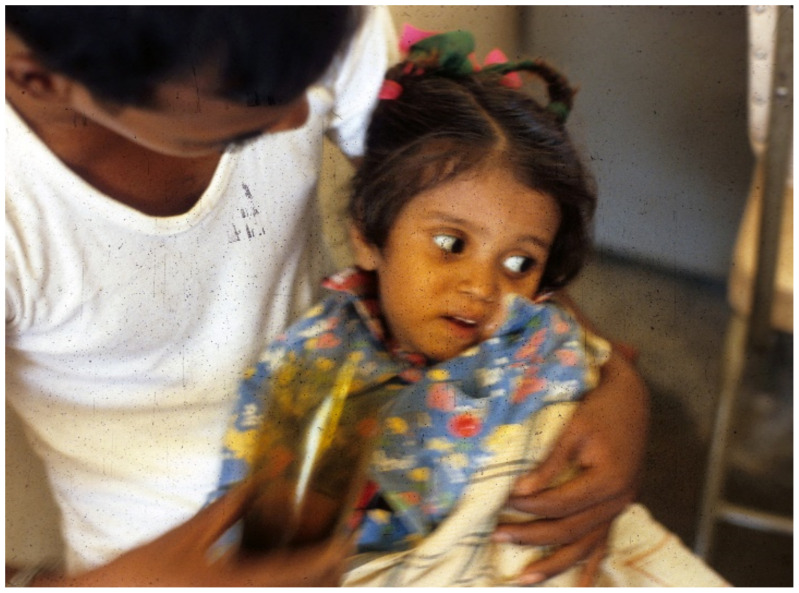
Patient now more alert, eyes less sunken at 1 h after starting ORS.

**Figure 6:**
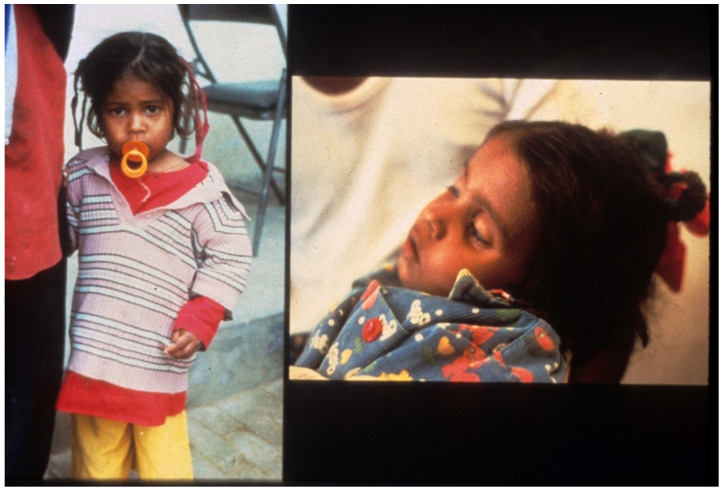
Patient after recovery, with pretreatment appearance on right.

**Figure 7:**
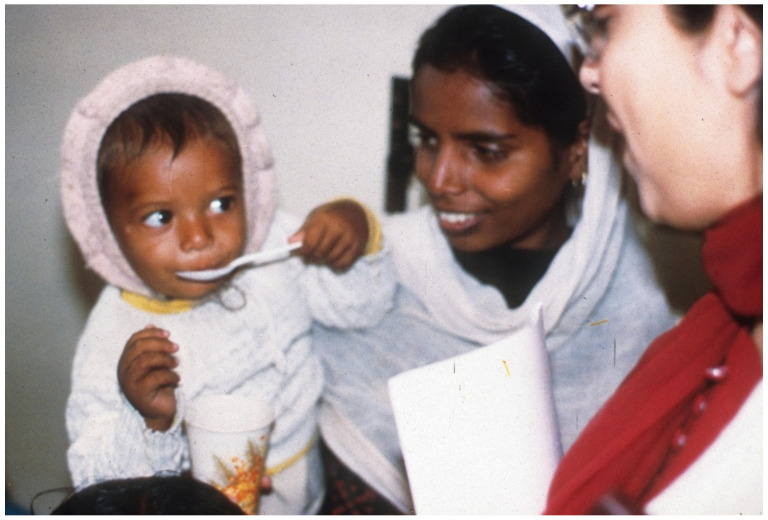
The ultimate goal: another child with acute watery diarrheas (AWD) starting ORS to prevent becoming dehydrated.

When no I.V. is available, patients need the early positive balance and superior retention this regimen affords, to *replace* pretreatment deficits and *maintain* positive water and electrolyte balance (Figure 1, Figure 2, Figure 3, Figure 4, Figure 5, Figure 6 and Figure 7).

## 4. The Evolution of ORT and ORS

The first successful clinical trial of ORT [17] was successful based on the combination of an appropriate ORS and, importantly, an effective *method* of administering the solution. The ORS formulation used had an electrolyte content approximating that of cholera diarrheal fluid losses, with sodium halfway between that of pediatric and adult cholera diarrhea fluid, and 110 mMol/L. glucose, without which the patients could not absorb the sodium, chloride and water essential to effective therapy.

ORS formulations using a range of sodium levels, chiefly 90 mEq/L, with glucose as the substrate and additional water intake (or breast milk feedings) permitted, have proven effective in treating dehydration in patients with cholera, nonvibrio cholera (caused by enterotoxigenic *Escherichia coli* and other organisms producing cholera toxin analogs) and non-cholera AWD including rotavirus diarrhea [23] and have decimated global under-five AWD mortality [24,25]. However, ORS formulations with Na^+^ content significantly below patients’ stool Na^+^ losses lead to negative sodium balance, Na^+^ depletion with hyponatremia and heightened risk of hyponatremic complications, whether combined with glucose, rice or other substrates [6,9].

The therapeutic method which proved essential [12,17] to overcoming the failures of earlier ORT trials [26] consisted of rapid initial correction of shock when present on admission, using I.V. rehydration. Oral therapy began *as soon as* shock was corrected, generally after administering I.V. fluid equivalent to 10% of admission body weight (in populations with BMIs (body mass index) below Western levels.) Initially, oral therapy was administered at a rate of 0.5 to 0.75 L/h in adults, based on body weight [17]. If the GNB (gut net balance: see PNGB above) monitored during the first 4 h indicated a fluid requirement greater than that estimated on admission, oral therapy was increased to match the volume of losses. Hydration status was monitored by checking plasma sp. gr. during the transition to oral maintenance. A rise over 1.030 was an indication for additional I.V. fluids to avoid progression to severe dehydration, estimation of hydration status based on clinical signs alone being less sensitive and more dependent on variable subjective criteria.

The ORS formulation used contained 120 mEq/L of Na^+^ (ORS 120), suitable for treating cholera and nonvibrio cholera patients. Using this method, total I.V. fluid requirements of cholera patients admitted in shock averaged 80% less than in controls and plasma Na^+^ remained normal. Patients not in severe shock were rehydrated and maintained in water and electrolyte balance using ORT alone without I.V. fluids [27,28]. Since cholera patients given effective adjunct antibiotic therapy [29], and most hospitalized non-cholera AWD patients, have steadily declining stool volume after treatment begins [13], closely matching ORS volume and composition imbibed in each sequential 4 or 6 h period to volume and composition of losses in the prior period by using fluids of appropriate composition ensures maintenance of PNGB of both water and electrolytes [12]. In the first large-scale field trial using this method [30], total I.V. requirements averaged 3.0 L in cholera patients arriving in shock whose average admission weight was 40 kg. Nonvibrio cholera patients not in shock on admission were successfully treated using glucose or glucose+glycine ORT alone with no I.V. fluids. Plasma sodium remained normal [28].

**Figure 8:**
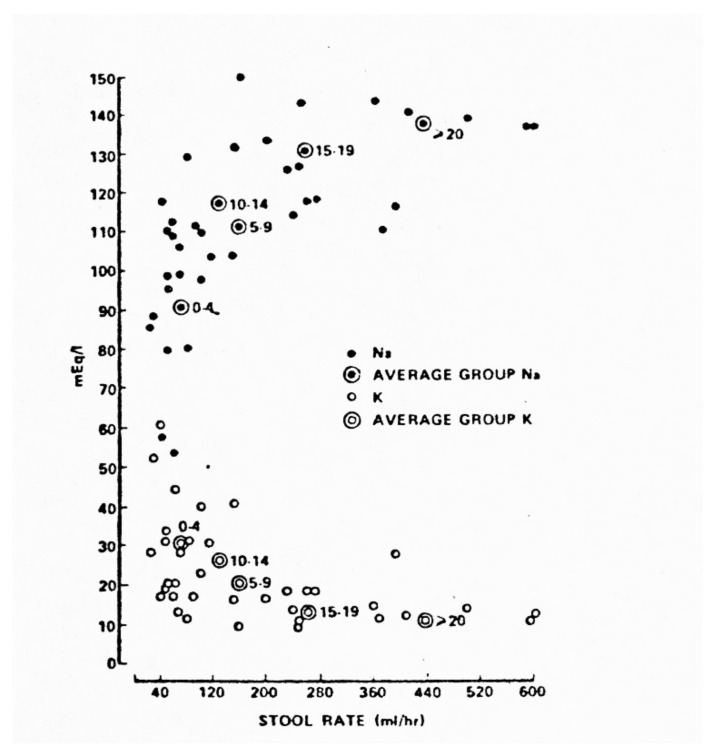
Relationship of diarrheal sodium and potassium losses (mEq/L) to stooling rate in 37 cholera patients during a period of maximum diarrhea 12–24 h after admission. At all ages, stool sodium tends to rise and potassium to fall at higher diarrhea rates. The numbers of patients were: 12 (0–4 yrs old), 10 (5–9 yrs), 6 (10–14 yrs), 2 (15–19 yrs), 7 (20 yrs and over). From Lancet, 30 October 1976, p. 957.

The ORS 90 formulation with 90 mEq/L of sodium (abbreviated “WHO 90” here because the Oralyte name has been copied by other ORS brands) was devised as a “compromise” between a formulation approximating the mean composition of cholera diarrhea fluid and that of noncholera pediatric AWDs. Since diarrheal stool content of sodium (directly) and potassium (inversely) correlate closely with diarrhea rate [31] (Figure 8) and cholera diarrhea rates are greatly in excess of average non-cholera AWD rates, the “compromise” provided excess sodium and insufficient potassium for pediatric non-cholera AWD patients and insufficient sodium for pediatric and adult cholera patients, if the method of matching fluid losses with equal volumes of ORS was used. After the initial clinical trial of the WHO ORS 90 formulation [32], it was noted [33] that, to maintain positive sodium balance using this formulation in cholera patients, the patients would have to drink an amount of the ORS equivalent to one and one-half times their diarrhea volume of the previous intake and output period, rather than simply matching that volume, in order to avoid negative Na^+^ balance and hyponatremia. Some patients would be unable to imbibe such large volumes over 24–32 h, leading to increased failure rates. However, the 1.5 X losses requirement was not promoted for general use, though it obviated the need for a separate ORS formulation for cholera. In research studies, however, it has been often matched or exceeded, somewhat confounding the conclusions regarding formulation impact *per se* [7,8,9,34].

The WHO 90 formulation was nonetheless highly successful in reducing global diarrheal mortality, since it was within the range of effective formulations suitable for most mild and moderate AWDs, in which ORS intake is limited by low diarrhea volume and short duration; any “extra” sodium in that formulation is needed to replace pretreatment losses when ORS 90 is used for both rehydration *and* maintenance without I.V. rehydration. In addition, allowance of extra free water (or breast milk [35]) given to pediatric AWD patients, either permissively (“ad libitum”) or in the fixed 2:1 ratio [36] prevented hypernatremia, the 2:1 method having the added safety factor of offering protection against instances of wrongly mixed hyperconcentrated ORS preparations. Also, the 90 mEq/L ORS proved safe and effective for treatment of noncholera AWDs in neonates and of children with hypo- or hypernatremia on admission when the 2:1 regimen was used [37]. The 2:1 regimen permitted use of the WHO 90 ORS packet in noncholera pediatric diarrhea patients and had the advantage of promoting early PNGB while avoiding transient hypernatremia [38].

In cholera patients, the inevitable high incidence of negative sodium balance, hyponatremia and, in some patients, seizures and other complications of hyponatremia, was overlooked until 2006 (10) due to lack of sodium balance studies plus inadequate safety surveillance for several decades in those areas where cholera was highly prevalent. The fundamental differences between cholera (including nonvibrio cholera) and other AWDs in magnitude of losses and pathophysiology underline the inferior efficacy for maintenance of Na^+^ balance and for avoidance of hyponatremia when ORS formulations with glucose or rice with ORS 90 or less are used to treat cholera patients.

## 5. Major Differences in Pathophysiology of Cholera and Non-Cholera AWDs

Recent studies have found that absorption of sugars and amino acids promoting active transport of sodium is not merely intact but is *increased* in response to cholera toxin [39,40,41,42,43,44,45]. This provides an explanation for the fact that oral therapy using ORS formulations with combined glucose and glycine [46,47], alanine [48] or glutamine [49] and other similar substrate combinations, and rice-based ORS (34) (furnishing glucose and amino acids on hydrolysis [50] are all capable of enhancing absorption and reducing diarrhea duration and volume in cholera and nonvibrio cholera but appear to ***have no such effect*** in noncholera AWD, particularly in children [51,52,53,54,55] and notably in rotavirus diarrhea [56], in which glucose ORS is effective [23], although glucose is often detectable in the stools. Rotavirus diarrhea may represent a distinct pathophysiology in which added glycine or other actively transported amino acid, or added rice, does not yield any significant advantage. The pathophysiology of this and of other noncholera AWDs may also limit absorption of amino acids by effects on villus function [57], as do some antibiotics used in ORS clinical trials [58]. In acute porcine viral enteritis, sodium-dependent alanine transport in the brush border membrane is reduced [59], suggesting one mechanism possibly explaining the lack of alanine, glycine or glutamine efficacy in human noncholera, notably viral, AWD.

In both the normal and the diarrhea-affected small bowel, sodium secretion into the lumen proceeds according to its ***chemical*** gradient, ***not*** the osmolar gradient per se. In the normal small bowel, osmoregulation of luminal contents is achieved by a combination of sodium excretion into the lumen according to the chemical gradient of sodium, and water absorption or secretion into the lumen according to the osmolar gradient. Animal studies have shown that cholera deranges normal small bowel osmoregulation due largely to interference with the absorptive component, with the effect that, in cholera, osmoregulation is accomplished solely by altering the rate of net *secretion* of water and salt. While rate of net water secretion into hypotonic lumenal solutions is reduced, rate of salt excretion is increased [60].

Repeated concern in the literature about the osmolality of ORS has persistently ignored the fact that the most successful ORS for cholera, containing glucose and glycine, has the *highest* osmolality of any successful ORS to date, proving that *absorbability trumps osmolality* as regards success rates of different ORS formulations. [46,47]. The same probably holds true for rice ORS, since the only available evidence indicates that rice, like sucrose, must be hydrolyzed in the intestinal lumen before the products of digestion are absorbed [61]. Rice is also reported to contain an antidiarrheal agent possibly interfering with adenyl cyclase [62,63], but such an agent would not be effective in diarrhoeal disorders related to other biochemical mechanisms, consistent with the clinical benefits of rice ORS being confined to cholera.

While reports are conflicting as to whether a rice (or rice product) diet has any additional effect on patients receiving ORS with glucose or other non-rice substrates [64,65], the effects of a rice diet on outcomes in patients receiving other ORS formulations have not been reported. However, it is counterintuitive, if rice has a positive beneficial effect, that giving a rice diet to patients receiving ORS without rice would have no effect.

In an effort to promote a single global ORS formulation, international bodies have recommended [1] a single ORS formulation with lower glucose, lower sodium (75 mEq/L.) and lower osmolality (“75 ORS”), based primarily on a modest and often clinically insignificant diminution in GDR, diarrhea duration and (variably in some studies) so-called “unscheduled” I.V. fluids, or I.V. fluids resumed post-rehydration, these modest advantages occurring *only* in noncholera pediatric populations, ***not*** in cholera patients [1].

An alternative mechanism explaining the apparent “benefits” of reduced diarrhea rates when hypoosmolar ORS is used is that the induction of hyponatremia by such formulations itself lowers diarrhea rate [66].

The “low glucose, low sodium, low osmolarity” ORS formulation is suboptimal for adults and children with cholera, because it contains sodium and chloride content far below that lost in cholera diarrhea. Use of the 75 ORS formulation in the majority of adult and pediatric cholera patients is, like WHO90, suboptimal in replacing sodium losses and causes even greater negative sodium balance with very large net sodium losses, which will lead to hyponatremia and, in a subset of cholera patients so treated, to seizures and other symptoms of severe hyponatremia.

Unlike the case with the WHO 90 ORS using the 2:1 regimen [34], the safety of ORS 75 in terms of net sodium balance when treating AWD in neonates or treating children with pre-existing hyponatremic or hypernatremic dehydration (a very high percentage of AWD patients at some centers) [67] has not been determined.

The assumption that cholera patients made hyponatremic rarely suffer adverse outcomes rests on no evidence, since, despite the magnitude of sodium losses using 75 ORS (Table 1), these have not been systematically looked for using established standardized tests of the well-known serious sequelae of hyponatremia [68,69,70] in any follow-up studies of hyponatremic pediatric or adult cholera patients.

Furthermore, the seizures seen in cholera patients made hyponatremic by use of this formulation have been arbitrarily attributed to other causes. For example, shigellosis is listed as a possible cause, whereas seizures are not a feature of shigellosis in the absence of hyponatremia [71]. Additionally, the “withdrawal” from analysis of patients with complicating other disorders transferred to the ICU [6,10] has the effect of obscuring the degree of harm caused by hyponatremia in the most vulnerable patients [72,73] who have a case fatality rate of 10% [74].

## 6. Limitations of Existing Studies

The Cochrane reviewed reports subdivided the international definition of hyponatremia (<135 mEq/L.) arbitrarily into cutoffs of 130, 127 or 125 mEq/L. of sodium [5,6,7,9,10], but, as previously noted: “neurocognitive deficits are evident, even in apparently asymptomatic patients, when such changes are specifically probed for.” Such deficits occur after hyponatremia of diverse causes in adults and children [68,69]. Detection of such deficits, which have been associated not only with seizures and stupor, but with less obvious signs and symptoms (e.g., headache, muscle cramps, weakness, restlessness, disorientation, depressed reflexes, gait disturbances, developmental retardation) require detailed examination and use of sensitive clinical tests, including the Mini-Mental State Examination, the Clock Completion test, the Audio Recording Cognitive Screening tool and a battery of attention tests (Visual Vigilance, Working Memory or Digit Span, Go/No Go, Intermodal Comparison, Divided Attention and Phasic Alert). No such screening was conducted in any of the Cochrane reviewed studies, whether in hyponatremic patients with or without seizures in hospital or in follow-up after discharge. The assumption stated in some reports indicating that dietary sodium should correct sodium deficits is by no means guaranteed and does not obviate the need to thoroughly check for hyponatremia-induced neurologic and developmental deficits. Furthermore, the assumption of dietary correction is particularly doubtful in severely malnourished diarrhea patients [67] and those with multiple AWD episodes per annum, which can number nine or more in some areas [75].

Unlike the case with the WHO 90 ORS using the 2:1 ratio [36], safety of ORS 75 in terms of net sodium balance when treating neonates or treating children with pre-existing hyponatremic or hypernatremic dehydration has not been tested. Safety studies over time in children with chronic hyponatremia or multiple AWD episodes annually are also needed.

Part of the confusion over this issue arises from the incomplete design of studies aiming to demonstrate “low” seizure rates in such patients, without follow-up for more long-term harmful effects. The only published large-scale survey compared seizure rates in cholera patients-treated chiefly (92%) with the low-sodium *rice* ORS to those treated in a prior year with the original glucose WHO 90 ORS. However as noted above, the WHO 90 formulation was significantly deficient in sodium compared to the sodium content of cholera diarrhea and was bound to cause negative sodium balance leading to hyponatremia in cholera patients unless, rather than simply matching their losses, they drank an amount equivalent to at least one and one-half times the volume of their diarrhea fluid, as originally noted soon after the formulation adopted by WHO was tested [31]. This would exceed drinking capacity in profusely purging patients, and the need to drink more of that formulation than with the earlier cholera ORS formulations in order to avoid hyponatremia was largely ignored. However in one Cochrane-reviewed study, remarkably, patients were given *twice* or *thrice* the amount of ORS to drink compared to the volume of their losses [7], a methodology placing a huge burden on patients and one sure to lead to increased failure rates in the field.

Other differences in methodology between study centers, confounding interpretation of ORS formulation effects, include the different quantities and duration of I.V. fluids given in the “rehydration” phase [5,6,7,8,9], percentage of severely dehydrated patients (4 vs. 23% [3]) or inclusion [4] vs. exclusion [3] of severely malnourished patients and identification [4,5,6,7,8,9] or lack thereof [3] of concurrent antibiotic therapy (erythromycin may inhibit jejunal D-galactase and sucrase [58]), omission of patient weights [3,4,7,10], inclusion or omission [7,8,9,10] of foods given during studies, of stool volume [4,10] or plasma sp. gr. measurements [3,5,6], use of different therapeutic methods (matching intake with output vs. giving a fixed number of ORS packets regardless of diarrhea duration [3,5]; allowing patients to become severely dehydrated after initial dehydration before resuming I.V. fluids [3,5,6], inclusion vs. exclusion of complete data on long-duration and high volume diarrhea patients [7], and reporting only 24 h serum Na^+^ levels in studies lasting 43–44 h [6].

Discrepant results include more unscheduled I.V. fluid needed in the low Na^+^ group [4,6] and highly variable disease severity (total stool volumes) between different centers’ outbreaks [3,4,5,6,7,8,9,10]. In one rice ORS study [8], stool volume fell 17% but inexplicably ORS volume fell 27%, suggesting lax management. These and other methodologic variations between centers in patient selection and management undoubtedly account for the considerable variability in results obtained. Many studies comparing different ORS formulations suffer from similar deficiencies and it is not surprising that conflicting results are not uncommon, chiefly from failure to prestratify by initial stool rates in most studies and use of different rehydration and maintenance methods and percent of breast-fed or food-fed infants.

In the Cochrane-reviewed studies, clinically estimated state of hydration (actually of severe dehydration) was used as the criterion for resuming I.V. fluids, called “unscheduled I.V.s”, but the data on specific clinical signs triggering I.V. resumption were omitted. The fact that in some studies severe dehydration could be permitted to recur [3,5,6] suggests a failure to respond to lesser degrees of recurrent dehydration or lapses in monitoring of the clinical signs of dehydration, which could have been more accurately and objectively accomplished by monitoring plasma sp. gr. levels.

Interpretation of results is also clouded by several other issues. Studies ostensibly comparing 75 with 90 mEq/L. formulations actually compared 75 or 90 mEq/L. Na^+^ given together with dietary rice preparations or noodles and salt [3,4,5,6,7,8,9,10]. Additionally, total quantities of I.V. fluids given after rehydration, representing additional salt loading offsetting sodium deficits, were not presented [3,4,5,6,7,8,9,10]. One study substituted stool frequency for stool volumes [4], but stool frequency can be high in low-volume inflammatory AWD, so the validity of this substitution is questionable, and frequency has not been correlated directly with total stool volume. The range of etiologic agents also differed between centers and seasons [3,4]. A claim that use of the lower Na^+^ ORS would reduce blood-borne diseases [6] is without any basis.

## 7. Discussion

Despite these limitations, most studies in pediatric or adult cholera patients concluded that the efficacy of 75 and 90 mEq/L. Na^+^ ORS formulations was similar, with neither ORS superior. Most studies showed no clinically or statistically significant differences in key parameters like total stool volume and duration, vomiting incidence and unscheduled I.V. rate. However, the obvious fact that the efficacy in terms of maintaining sodium balance was inadequate in both groups went unstated.

Since both the 75 and the 90 mEq/L. sodium ORS glucose formulations lead to hyponatremia when given to cholera patients losing 100–145 mEq/L of sodium, it was to be expected that the *rates* of hyponatremia would be the same using either of the two formulations. In that light, the comparisons of hyponatremia rates using 75 ORS vs. 90 ORS to show no significant differences was a straw-man hypothesis. The unmeasured net *negative sodium balance* will certainly be greater using the 75 than using the 90 mEq/L. ORS formulation (see Table 1 above).

Surprisingly, from both the efficacy and safety perspectives, not a single sodium balance study was conducted prior to promoting the low-sodium glucose (or rice)-based ORS formulations for use in cholera patients, and none has appeared since. No sufficiently sized and powered properly controlled sodium balance efficacy and safety study comparing results with ORS containing 75 vs. 90 mEq/L. Na+ has been done with either rice or glucose as substrates. Two such studies formerly said to be planned (2, see pp. 34–35 for studies #NCDT00490932 and NCT 00672308) apparently have not been published, if indeed completed as reported [2].

The danger of profound iatrogenic sodium losses and hyponatremia complications resulting from treatment of pediatric and adult cholera patients treated with the 75 mEq/L. ORS sodium formulation will be even more pronounced when treating patients harboring antibiotic resistant *V. cholerae*, who may need up to 100 L of ORS to replace their stool losses after initial I.V. rehydration [76].

Serum sodium in adults does not decline until there is more than 200 mEq net sodium loss. Monitoring only serum sodium does not give a correct estimate of total body sodium loss. Cholera patients of all ages have massive sodium losses using the low-sodium ORS, leading to serum sodium declining to hyponatremic levels in >50% of adult cholera patients treated with ORS 90 with glucose (Nalin D, unpublished data). This results in a cutoff of antidiuretic hormone (ADH) with resulting polyuria even during dehydration, and this has been misinterpreted in clinical studies of low-sodium ORS as a sign of good hydration [5,7] which it is not. This exemplifies the clinical misinterpretation resulting from the polyuria, which use of the low-sodium solution can lead to.

In the settings of rural cholera treatment centers or home treatment, management of hyponatremic seizures and related complications is likely to have serious consequences which are attributable only to the use of this formulation instead of one matching more closely that of cholera diarrhea. Such a formulation should contain 120 mEq/L. of sodium, providing that ORS substrates, which in cholera alone enhance salt absorption more than glucose alone, are used in the ORS, including formulations with glucose plus glycine or rice powder (which yields glucose, amino acids and antidiarrheal components on hydrolysis [50,62,63]). The price of glycine has been mistakenly mentioned as a reason not to use it for cholera patients, but in fact glycine and glucose are available in bulk as food additives on the global wholesale market at about the same price, such as $1/kg [77,78]. Other amino acids which also promote active transport are far costlier. Rice or glucose plus glycine ORS packets would also offer savings in reduced hospitalization time; whether rice or glucose plus glycine ORS has any advantage in terms of commercial packet shelf life, and whether all of the many varieties of rice are equally effective, have not been determined. Results obtained using rice ORS or glycine–glucose ORS have been generally similar in that the advantages with either ORS are reproducible only in studies with a majority of cholera and/or nonvibrio cholera patients, not in patients with AWDs of other etiologies. This again underlines the pathophysiologic peculiarities of diarrhea-caused *V. cholerae* or by strains of enterotoxigenic *E. coli* and related pathogens producing cholera toxin analogs, versus other diarrheal pathogens.

Rice is also ineffective in patients with rice carbohydrate malabsorption [79], in which boiled rice fed to children with cholera leads to increased volume and duration of diarrhea [80]. Lastly, a Cochrane review concluded that the advantages of rice ORS, like those of glycine–glucose ORS, are seen in cholera or nonvibrio cholera but not in other types of AWD [81].

## 8. Conclusions

In sum, fear of what has turned out to be chiefly transient mild or moderate hypernatremia has led to ORS formulations inducing high prevalence hyponatremia, notably at a time when pediatric recommendations for intravenous fluid therapy have shifted to higher sodium I.V. solutions [82,83,84,85]. Ironically, hypernatremic seizures, feared when glucose ORS with 90 mEq/L. Na^+^ is used in noncholera AWD patients, appeared in only 1 of 48,511 WHO 90-treated patients surveyed in the largest series, compared with 47 hyponatremic seizures [10]. The incidence rates reported in that study were minimized by using a denominator including a majority of nondehydrated or mildly dehydrated patients not in the risk pool for hyponatremia. When the denominator was restricted to severely dehydrated patients, the hyponatremic seizure rate was 0.15% in the study group, but the comparable rate in the comparison group was omitted. In another study [3], one out of every 13 children with serum Na^+^ < 125 had seizures. Projected on a global basis, this represents a very significant morbidity burden linked with this formulation.

The dichotomy in efficacy of the low Na^+^ ORS formulation between cholera and non-cholera AWDs presents a paradox: in cholera, the goal of an ORS with 120 mEq/L. Na+ and either rice or glycine–glucose, which significantly reduce both duration and volume of diarrhea safely and without profound net sodium losses, is an attractive option. In noncholera pediatric AWDs, the ORS with 3 lowered parameters appears to offer similar benefits but has inferior efficacy for maintaining sodium balance and leads to an iatrogenic increased incidence of hyponatremic toxicity when used for cholera. Perhaps studies in which less than three variables are changed would be useful. In cholera, 75 ORS with rice also causes hyponatremia [9].

Outcomes of ORS formulation studies are etiology-dependent, cholera and related diseases benefiting very significantly from glucose ORS with added actively transport-promoting amino acids, benefits not seen in rotavirus and related noncholera AWDs in which absorption of glucose is sufficient for successful ORT [23], but absorption of added amino acids is evidently blocked by pathogenetic factors.

Cholera outbreaks have occurred in recent years in Haiti, Yemen and many African countries and are quickly recognized. A choice is at hand between two different oral treatment modalities for cholera, an ORS with 120 mEq/L. Na^+^ plus rice or glucose–glycine, vs. one using 75 ORS with glucose or rice. The 75 ORS option is significantly less effective in maintaining sodium balance and has a less favorable though inadequately monitored safety profile. No clinical trials to date have employed standard sensitive neurologic tests [68,69] to monitor for adverse effects of hyponatremia other than seizures, including long-term effects on developmental parameters and delayed mortality. If 75 ORS is to be promoted for cholera, its safety profile should be firmly established as indicated in Table 2.

It is in the long-term public health interest to choose the safer and more effective ORS formulation for cholera. Even a “low” percentage of hyponatremic seizures and other neurologic and developmental sequelae translates globally into thousands of cases annually, a major avoidable morbidity. The time has come to recognize that two different ORS formulations are needed, one with rice or with glucose plus glycine for use in cholera epidemics, and one for noncholera AWDs. Both rice and glycine–glucose ORS have advantages in cholera, but for use in packets glycine, which does not require boiling, may be advantageous, and may have superior shelf life before and after mixing [86], while preserving the savings in reduced hospitalization time for patients at cholera treatment centers when either ORS is used.

However, if glucose-ORS alone is to be globally recommended and if the programmatic goal of promoting only a single ORS packet is the overweening concern, another possible alternative meriting clinical trials would be to alter the volume of water used to dilute the ORS packets when confronting cholera. For example, an ORS suitable for use in cholera can be made by reconstituting four WHO 90 packets in 3 L of potable water (Table 3). The dilutional water volume can easily be measured, as now, using household containers, or standardized by use of calibrated plastic bags [87]. A similar solution has been found suitable for use in hospitalized cholera patients of all ages when the matching method is used to balance intake with output [88]. The resulting glucose content is close to that found optimal in early balance studies [32].

## Figures and Tables

**Table 1:** Comparison of calculated net sodium losses in cholera patients treated with ORS containing 75 vs. 90 mEq/L. sodium. The 8 L total stool volume is an example based on figures in J. Pediatrics 78: 355–358, 1971. * Na^+^ in mEq. Stool sodium levels from Table 11-2, P.225, in Cholera, Eds. Barua and Burrows 1974. W.B. Saunders Co., Philadelphia.

Na^+^ Losses in Cholera Patient Given (ORS75 vs. ORS90)		
	ADULTS	CHILDREN
Stool Na^+^ (mEq/L)	140	101
ORS Na^+^	75 vs. 90	75 vs. 90
Na^+^ Loss	−65 vs. −50	−26 vs. −10
Stool Vol. (L)	25	8
Total Na^+^ loss (mEq) *	−1625 vs. −1250	−208 vs. −80

**Table 2:** Comprehensive safety studies recommended to fully assess safety of hyponatremia induced or aggravated in cholera patients receiving ORS 75. For standard tests, see [68,69].

Recommended Safety Studies of 75 ORS in Cholera Patients	
STUDIES	PURPOSE
Na^+^ Balance	Determine size of Na^+^ deficite
Clinical Sequelae	R/O acute sequelae using standard tests
Follow-up Studies	R/O developmental deficits and excess post-convalescent mortality

**Table 3:** How 4 ORS 90 packets could be dissolved in 3 L of water to make a solution more suitable for replacing cholera patients’ electrolyte losses. Using ORS 75 packets with 2.6 g NaCl each, a similar solution could be prepared by dissolving four packets in 2.5 L of water.

ORS Suitable for Cholera Patients	
Dissolve 4 Packets of 90 ORS in 3 L Water	Resulting ORS Concentrations
	Na^+^ 120 *
	K^+^ 27 *
	Cl^−^ 107 *
	Citrate 13@
	Glucose 147@

* mEq/L, @mMol/L.

## Abbreviations

AWD	Acute Watery Diarhhea
ORS	Oral Rehydratation Solution
ORT	Oral Rehydratation Therapy
ORS75	ORS with 75 mEq/L Sodium (Na^+^)
ORS90	ORS With 90 mEq/L Sodium (Na^+^)
ORS120	ORS With 120 mEq/L Sodium (Na^+^)
GDR	Gross Diarrhea Rate, ml/hr
NGB	Net Gut Balance (oral intake) − (stool+vomitus) in Liters
PNGB	Positive Net Gut Balance
CHOLERA	AWD Caused by *Vibrio cholerae*
ETEC	Enterotoxigenic *Escherichia coli*
NONVIBRIO CHOLERA	Severe AWD Due to ETEC or Bacteria with Cholera Toxin Analogs

## References

[1] WHO Reduced Osmolarity Oral Rehydration Salts (ORS) Formulation; Department of Child and Adolescent Health and Development. https://apps.who.int/iris/handle/10665/67322.

[2] Musekiwa A., Volmink J., Cochrane Infectious Diseases Group (2011). Oral Rehydration Salt Solution for Treating Cholera: ≤270 mOs/L solutions vs. ≤310 mOsm/L Solutions. Cochrane Database Syst. Rev..

[3] CHOICE Study Group (2001). Multicenter, randomized, double-blind clinical trial to evaluate the efficacy and safety of a reduced osmolarity oral rehydration salts solution in children with acute watery diarrhea. Pediatrics.

[4] Alam S., Afzal K., Maheshwari M., Shukla I. (2000). Controlled trial of hypo-osmolar versus world Health Organization Oral rehydration solution. Indian Pediatrics.

[5] Pulungsih S.P., Punjabi N.H., Rafli K., Rifajati A., Kumala S., Simanjuntak C.H., Yuwono Lesmana M., Subekti D., Sutoto, Fontaine O. (2006). Standard WHO_ORS versus reduced-osmolarity ORS in the management of cholera patients. J. Health Popul. Nutr..

[6] Alam N.H., Majumder R.N., Fuchs G.J., The CHOICE study group (1999). Efficacy and safety of oral rehydration solution with reduced osmolarity in adults with cholera: A randomized double-blind clinical trial. Lancet.

[7] Faruque A.S.G., Mahalanabis D., Hamadani J.D., Zetterstrom R. (1996). Reduced osmolarity oral rehydration salt in cholera. Scan. J. Infect Dis..

[8] Bhattacharya M.K., Bhattacharya S.K., Dutta D., Deb A.K., Deb M., Dutta A., Saha C.A., Nair G.B., Mahalanabis D. (1998). Efficacy of oral hyposmolar glucose-based and rice-based oral rehydration salt solutions in the treatment of cholera in adults. Scan. J. Gastroent..

[9] Dutta M.K., Bhattacharya M.K., Deb A.K., Sarkar A., Chatterjee A., Biswas A.B., Chatterjee K., Nair G.B., Bhattacharya S.K. (2000). Evaluation of oral hypo-osmolar glucose-based and rice-based oral rehydration solutions in the treatment of cholera in children. Acta Paediatr..

[10] Alam N.H., Yunus M., Faruque A.S.G., Gyr N., Sattar S., Parvin S., Ahmed J.U., Salam M.A., Sack D.A. (2006). Symptomatic hyponatremia during treatment of dehydrating diarrheal disease with reduced osmolarity oral rehydration solution. JAMA.

[11] Phillips R.A. (1968). Asiatic cholera. Ann. Rev. Med..

[12] Nalin D.R. (1987). Oral therapy for diarrheal diseases. J. Diarrheal Dis. Res..

[13] Hirschhorn N., Kinzie J.I., Sachar D.B., Northrup R.S., Taylor J.O., Ahmad S.Z., Philllips R.A. (1968). Decrease in net stool output in cholera during intestinal perfusion with glucose-containing solutions. NEJM.

[14] Nalin D.R., Levine M.M., Hornick R., Bergquist E., Hoover D., Holley H.P., Waterman D., Van Blerk J., Matheny S., Sotman S. (1979). The problem of emesis during oral glucose-electrolytes therapy given from the onset of severe cholera. Trans. Roy. Soc. Trop. Med. Hyg..

[15] Cash R.A., Nalin D.R., Toaha K.M.M., Huq Z. (1969). Acetate in the correction of acidosis secondary to diarrhea. Lancet.

[16] Harvey R.M., Enson Y., Lewis M.L., Greenough W.B., Ally K., Panno R.A. (1968). Hemodynamic studies on cholera, Effects of hypovolemia and acidosis. Circulation.

[17] Nalin D.R., Cash R.A., Islam R., Molla M., Phillips R.A. (1968). Oral maintenance therapy for cholera in adults. Lancet.

[18] El-Mougi M., Hendaw A., Koura H., Hegazi E., Fontain O., Pierce N.F. (1996). Efficacy of standard glucose-based and reduced-osmolarity maltodextrin-based oral rehyration solutions: Effect of sugar malabsorption. Bull. WHO.

[19] Lifschitz F., Coello-Ramirez P., Gutierrez L.L.C. (1970). Monosaccharide intolerance and hypoglycemia in infants with diarrh74, 471-477.ea: Metabolic studies in 23 infants. J. Peds.

[20] Hirschhorn N., Nalin D.R., Cash R.A. (2002). CHOICE Study Group Trial. Pediatrics.

[21] Saniel M.C., Zimicki S., Carlos C.C., Maria A.C.S., Balis A.C., Malacad C. (1997). J. Diarrhoeal Dis. Res..

[22] Isolauri E. (1985). Evaluation of an oral rehydration solution with Na+ 60 mmol/L. in infants hospitalized for acute diarrhea or treated as outpatients. Acta Paediatr. Scand.

[23] Nalin D.R., Levine M.M., Mata L., de Cespedes C., Vargas W., Lizano C., Loria A.R., Simhon A., Mohs E. (1979). Oral rehydration and maintenance of children with rotavirus and bacterial diarrheas. Bull. WHO.

[24] Snyder J.D., Merson M.H. (1982). The magnitude of the problem of acute diarrheal disease: A review of active surveillance data. Bull. WHO.

[25] GBD 2016 Diarrhoeal Disease Collaborators (2018). Estimates of the global, regional and national morbidity, mortality and aetiologies of diarrhea in 195 countries: A systematic analysis for the Global Burden of Disease Study 2016. Lancet.

[26] Ruxin J. (1994). Magic Bullet: The history of oral rehydration therapy. Med. Hist..

[27] Cash R.A., Nalin D.R., Forrest J., Abrutyn E. (1970). Rapid correction of the acidosis and dehydration of cholera with an oral solution. Lancet.

[28] Nalin D.R., Cash R.A. (1970). Oral or nasogastric maintenance therapy for diarrheas of unknown etiology resembling cholera. Trans. Roy. Soc. Trop. Med. Hyg..

[29] Greenough W.B., Gordon R.S., Rosenberg I.S., David B.I., Benenson A.H. (1964). Tetracycline in the treatment of cholera. Lancet.

[30] Cash R.A., Nalin D.R., Rochat R., Reller B., Haque E., Rahman M. (1970). A clinical trial of oral therapy in a rural cholera treatment center. Am. J. Trop. Med. Hyg..

[31] Nalin D.R., Cash R.A. (1976). Sodium content in oral therapy for diarrhea. Lancet.

[32] Pierce N.F., Banwell J.G., Mitra R.C., Caranasos G.J., Keimowitz R.I., Mondal A., Manji P.M. (1968). Effect of intragastric glucose-electrolyte infusion upon water and electrolyte balance in Asiatic cholera. Gastroenterology.

[33] Nalin D.R. (1970). Oral Cholera Therapy. Ann. Intern. Med..

[34] Molla A.M., Ahmed S.M., Greenough W.B. (1985). Rice-based oral rehydration solution decreases the stool volume in acute diarrhea. Bull. WHO.

[35] Roy S.K., Rabbani G.H., Black R.E. (1984). Oral rehydration solution safely used in breast-fed children without additional water. J. Trop. Med. Hyg..

[36] Nalin D.R., Levine M.M., Mata L., de Cespedes C., Vargas W., Lizano C., Loria A.R., Simhon A., Mohs E. (1978). Comparison of sucrose with glucose in oral therapy of infant diarrheas. Lancet.

[37] Pizarro D., Possada G., Villavicencio N., Mohs E., Levine M.M. (1983). Hypernatremic and hyponatremic diarrheal dehydration. Treatment with oral glucose-electrolyte solution. Am. J. Dis. Child..

[38] Nalin D.R., Harland E., Ramlal A., Swaby D., McDonald J., Gangarosa R., Levine M., Akierman A., Antonine M., Mackenzie K. (1980). Comparison of low and high sodium and potassium content in oral rehydration solutions. J. Pediatr..

[39] Clancy B.M., Czech M.P. (1990). Hexose transport stimulation and membrane redistribution of glucose transporter isoforms in response to cholera toxin, dibutyryl cyclic AMP and insulin in3T3-L1 adipocytes. J. Biol. Chem..

[40] Nath S.K., Rautureau M., Heyman H. (1989). Emergence of Na+-glucose cotransport in an epithelial secretory cell line sensitive to cholera toxin. Am. J. Physiol..

[41] Moule S.K., Bradford N.M., McGivan J.D. (1987). Short-term stimulation of Na+-dependent amino acid transport by dibutryl cyclic AMP in hepatocytes. Characteristics and partial mechanism. Biochem. J..

[42] Tai Y.H., Perez E., Desjeux J.F., Alverado F., Van Os C.H. (1986). Cholera toxin and cyclic AMP stimulate D-glucose absorption in rat ileum. Ion Gradient-Coupled Transport.

[43] Wright Em Hirsh J.R., Loo D.D., Zampighi G.A. (1997). Regulation of Na+/glucose cotransporters. J. Exp. Biol..

[44] Flach C.F., Lange S., Jennische E., Lonnroth I. (2004). Cholera toxin induces expression of ion channels and carriers in small intestinal mucosea. FEBS Lett..

[45] Schiller L.E., Santa Ana C., Porter J., Fortran J.S. (1997). Glucose-stimulated sodium transport by the human intestine during experimental cholera. Gastroenterology.

[46] Nalin D.R., Cash R.A., Rahaman M., Yunus M. (1970). Effect of glycine and glucose on sodium and water absorption in patients with cholera. Gut.

[47] Patra F.C., Mahalanabis D., Jalan K.N., Sen A., Banerjee P. (1984). In search of a super solution:controlled trial of glycine-glucose oral rehydration solution in infantile diarrhea?. Acta Pediatr. Scand.

[48] Patra F.C., Sack D.A., Islam A., Alam A.N., Mazumder R.N. (1989). Oral rehydration formula containing alanine and glucose for treatment of diarrhea: A controlled trial. BMJ.

[49] Punjabi N.H., Kumala S., Rasidi C., Witham N.D., Pulungsih S.P., Rivai A.R., Sukri N., Burr D.H., Lesmana M. (1991). Improving the ORS: Does glutamine have a role?. Am. J. Trop. Med. Hyg..

[50] Amankwah E.N., Adu E., Barimah V.M.J., Van Twisk C. (2015). Amino acid profiles of some varieties of rice, soybean and groundnut grown in Ghana. J. Food Process. Technol..

[51] Vesikari T., Isolauri E. (1986). Glycine supplemented oral rehydration solutions for diarrhea. Arch. Dis. Child..

[52] Pizarro D., Levine M.M., Posada G., Sandi L. (1988). Comparison of glucose/electrolyte and glucose/glycine/electrolyte oral rehydration solutions in hospitalized children with diarrhea in Costa Rica. J. Pediatr. Gastroenterol. Nutr..

[53] Ribiero H.D.C., Lifshitz F. (1991). Alanine-based oral rehydration therapy for infants with acute diarrhea. J. Pediatr..

[54] Gutierrez C., Villa S., Mota F.R., Calva J.J. (2007). Does an L-glutamine-containing, glucose–free, oral rehydration solution reduce stool output and time to rehydrate in children with acute diarrhea? A double-blind randomized clinical trial. J. Health Popul. Nutr..

[55] Gore S.M., Fontaine O., Pierce N. (1992). Impact of rice based oral rehydration solution on stool output and duration of diarrhea:meta-analysis of 13 clinical trials. BMJ.

[56] Mahalanabis D., Faruque A.G., Hoque S.S., Faruque S.M. (1995). Hypotonic oral rehydration solution in acute diarrhea: A controlled clinical trial. Acta Pediatr..

[57] Davidson G.P., Barnes G.L. (1979). Structural and functional abnormalities of the small intestine in infants and young children with rotavirus enteritis. Acta Paediatr..

[58] Navarro H., Arruebo M.P., Alcalde A.I., Sorribas V. (1993). Effect of erythromycin on D-galactose absorption and sucrose activity in rabbit jejunum. Can. J. Physiol. Pharmacol..

[59] Rhoads J.M., MacLeod R.J., Hamilton J.R. (1989). Diminished brush border membrane sodium-dependent L-alanine transport in acute viral enteritis in piglets. J. Pediatr. Gastroenterol. Nutr..

[60] Nalin D.R., Ally K., Hare K., Hare R. (1972). Effects of cholera enterotoxin on jejunal osmoregulation of mannitol solutions in dogs. J. Infect. Dis..

[61] Gray G.M., Ingelfinger F.J. (1965). Intestinal absorption of sucrose in man: The site of hydrolysis and absorption. JCI.

[62] Mathews C.J., MacLeod R.J., Zheng S.X., Hanrahan J.W., Bennett H.P., Hamilton J.R. (1999). Characterization of the inhibitory effect of boiled rice on intestinal chloride secretion in guinea pig crypt cells. Gastroenterology.

[63] Macleod R., Bennett H., Hamilton J. (1995). inhibition of intestinal secretion by rice. Lancet.

[64] Alam V.A., Ahmed T., Khatum, Molla A.M. (1992). Effect of food with two or four rehydration therapies: A randomized controlled clinical trial. Gut.

[65] Santosham M., Fyad I., Hashem M., Goepp J.G., Refaf M., Sack B. (1990). A comparison of rice-based oral rehydration solute and early feeding for the treatment of acute diarrhea in infants. J. Pediatr..

[66] Clarke A.M., Miller M., Shields R. (1967). Intestinal transport of sodium, potassium and water in the dog during sodium depletion. Gastroenteritis.

[67] Houston K.A., Gibb J.G., Maitland K. (2017). Oral rehydration of malnourished children with diarrhea: A systematic review (version 3). Wellcome Open Res..

[68] Arieff A.I., Ayus J.C., Fraser C.I. (1992). Hyponatremia and death or permanent brain damage in healthy children. BMV.

[69] Rondon-Berrios H., Berl T. (2015). Mild chronic hyponatremia in the ambulatory setting: Significance and management. Clin. J. Am. Soc. Nephrol..

[70] Shahrin L., Chistri M.J., Huq S., Nishath T., Christy M.D., Hannan A., Ahmed T. (2016). Clinical manifestations of hyponatremia and hypernatremia in under-five diarrheal children in a diarrhea hospital. J. Trop. Pediatr..

[71] Khan W.A., Dhar U., Salam M.A., Griffiths J.K., Rand W., Bennish M.L. (1999). Central nervous system manifestations of childhood shigellosis: Prevalence, risk factors and outcome. Pediatrics.

[72] Mitra A.K., Khan M.R., Alam A.N. (1991). Complications and outcome of disease in patients admitted to the intensive care unit of a diarrhoeal diseases hospital in Bangladesh. Trans. Roy. Soc. Trop. Med. Hyg..

[73] Chisti M.J., Pietroni M.A., Smith J.H., Bardhan P.K., Salam M.A. (2011). Predictors of death in under-five chidren with diarrhea admitted to a critical care ward in an urban hospital in Bangladesh. Acta Paediatr..

[74] Samadi A.R., Wahed M.A., Islam M.R., Ahmed S.M. (1983). Consequences of hyponatraemia and hypernatraemia in children with acute diarrhea in Bangladesh. BMJ Clin. Res. Ed..

[75] Sazawal S., Black R.E., Bhan M.K., Bhandari N., Sinha A., Jalla S. (1995). Zinc supplementation in young children with acute diarrhea in India. NEJM.

[76] Phillips R.A. (1966). Cholera in the perspective of 1966. Ann. Int. Med..

[77] www.alibaba.com/amino-acid-glycine-price.

[78] www.alibaba.com/glucosepowder.

[79] Khin-Maung-U, Bolin T.D., Duncombe V.M., Myo-Khin, Nyunt-Nyunt-Wai, Pereira S.P., Linklater J.M. (1992). Epidemiology of small bowel bacterial overgrowth and rice carbohydrate malabsorption in Burmese (Myanmar) village children. Am. J. Trop. Med. Hyg..

[80] Khinmaungu, Nyuntnyuntwai, Myokhin, Mumukhin, Tinu, Thanetoe (1986). Effect of boiled-rice feeding in childhood cholera on clinical outcome. Hum. Nutr. Clin. Nutr..

[81] Fontaine O., Gore S.M., Pierce N.F. (1998). Rice-based oral rehydration solution for treating diarrhea. Cochrane Database Syst. Rev..

[82] Eldridge D., Ledoux M. (2014). Needs more salt: Old hydration habits are hard to break. Lancet.

[83] Duke T., Molyneux E.M. (2003). Intravenous fluids for seriously ill children: Time to reconsider. Lancet.

[84] McNab S., Duke T., South M., Babl F.E., Lee K.j., Arnup S.J., Young S., Turner H., Davidson A. (2015). 140 mmol/L of sodium versus 77 mmol/L. of sodium in maintenance intravenous fluid therapy for children in hospital (PIMS): A randomized controlled double-blind trial. Lancet.

[85] Friedman J.N., Beck C.E., DeGroot J., Geary D.F., Sklansky D.J., Freedman S.B. (2015). Comparison of isotonic and hypotonic intravenous maintenance fluids: A randomized clinical trial. JAMA Pediatr..

[86] Bhattachariya S.K., Dutta P., Dutta D., Chakraborti M.K. (1990). Super ORS. Indian J. Public Health.

[87] Nalin D.R. (1978). A spoonful of sugar. Lancet.

[88] Nalin D.R., Cash R.A. (1970). Oral or nasogastric maintenance for cholera patients in all age groups. Bull. WHO.

