# Correction: Nalin, D. Issues and Controversies in the Evolution of Oral Rehydration Therapy (ORT). *Trop. Med. Infect. Dis.* 2021, *6*, 34

**DOI:** 10.3390/tropicalmed7060103

**Published:** 2022-06-14

**Authors:** David Nalin

**Affiliations:** Albany Medical College, Albany, NY 12208-3478, USA; nalindavid@gmail.com

**Error in Table**

In the original publication [1], an error appears in Table 3 in the original article, in which the number 75 incorrectly appeared in the left hand column instead of the correct number 90. The corrected Table 3 appears below. The authors apologize for any inconvenience caused and state that the scientific conclusions are unaffected. The original publication has also been updated.

## Figures and Tables

**Table 3:** How 4 ORS 90 packets could be dissolved in 3 L of water to make a solution more suitable for replacing cholera patients’ electrolyte losses. Using ORS 75 packets with 2.6 g NaCl each, a similar solution could be prepared by dissolving four packets in 2.5 L of water.

ORS Suitable for Cholera Patients	
Dissolve 4 Packets of 90 ORS in 3 L Water	Resulting ORS Concentrations
	Na^+^ 120 *
	K^+^ 27 *
	Cl^−^ 107 *
	Citrate 13@
	Glucose 147@

* mEq/L, @ mMol/L.
